# A Pregnant Woman with Primary Hyperparathyroidism Who Underwent Parathyroidectomy in the Second Trimester of Her Third Pregnancy: A Case Report

**DOI:** 10.24546/0100495980

**Published:** 2025-05-30

**Authors:** MASAYUKI TANAKA, HITOMI IMAFUKU, IROHA KUBOTA, KEITARO YAMANAKA, SONOKO SUDA, NAOHISA MASUKO, AKIKO UCHIDA, HIDENORI FUKUOKA, MASANORI TESHIMA, KAZUMICHI FUJIOKA, MASASHI DEGUCHI, KENJI TANIMURA, YOSHITO TERAI

**Affiliations:** 1 Department of Obstetrics and Gynecology, Kobe University Graduate School of Medicine, Kobe, Japan; 2 Division of Diabetes and Endocrinology, Kobe University Hospital, Kobe, Japan; 3 Department of Otolaryngology-Head and Neck Surgery, Kochi Medical School, Kochi, Japan; 4 Department of Pediatrics, Kobe University Graduate School of Medicine, Kobe, Japan

**Keywords:** Fetal growth restriction, Hyperparathyroidism, Intrauterine fetal death, Parathyroidectomy, Pregnancy

## Abstract

Primary hyperparathyroidism (PHPT) during pregnancy is rare, causing severe pregnancy complications. We report a pregnant woman with PHPT and a pregnancy complication history who underwent a single-gland parathyroidectomy during the second trimester and delivered at term. A 28-year-old pregnant woman, gravida 3, para 2, was referred to our hospital at 9 gestational weeks (GWs) because of previous intrauterine fetal death, preterm birth, and fetal growth restriction. She was diagnosed with PHPT during her second pregnancy, and her baby experienced neonatal hypocalcemia. However, she received no PHPT treatment afterward. Laboratory tests and neck ultrasound revealed hyperparathyroidism and an enlarged right superior parathyroid gland. She underwent a right superior parathyroidectomy at 24 GWs. She delivered a 2,136 g (−1.74 SD) healthy female infant at 37 GWs, and her baby demonstrated no neonatal hypocalcemia. Parathyroidectomy, even during pregnancy, should be considered among pregnant women with PHPT having a pregnancy or neonatal complication history.

## INTRODUCTION

Primary hyperparathyroidism (PHPT) is one of the leading causes of hypercalcemia, and its symptoms include fatigue, depression, polyuria, anorexia, and muscle weakness. Furthermore, PHPT during pregnancy is very rare and causes not only pregnancy complications, such as preterm delivery, fetal growth restriction (FGR), and intrauterine fetal death (IUFD), but also neonatal complications, including tetany and permanent hypoparathyroidism. An estimated 67% and 80% of the prevalence of pregnancy and neonatal complications, respectively, were observed in pregnant women with PHPT (1).

Several reports described parathyroidectomy for PHPT during pregnancy (2–4). We hereby report a case of a pregnant woman with PHPT who had one IUFD and one premature birth history, both with FGR, but who underwent a single-gland parathyroidectomy in the second trimester of her third pregnancy and successfully delivered a live newborn at term.

## CASE REPORT

This case report adhered to the principles of the Declaration of Helsinki. Written informed consent was obtained from the patient, and patient anonymity was maintained.

A 28-year-old pregnant woman, gravida 3, para 2, was referred to Kobe University Hospital at 9 + 2/7 gestational weeks (GWs) because of a pregnancy complication history. Her first pregnancy ended in IUFD with FGR at 24 GWs, and her second pregnancy ended in preterm delivery caused by FGR at 30 GWs.

Both of her previous pregnancies were managed at our university hospital. She underwent a checkup to determine the cause of FGR three months after her stillbirth. The results detected no antiphospholipid antibodies but only low protein S (PS) activity (49.7% [normal: 63.5%–149.0%]).

She was diagnosed with FGR at 26 + 4/7 GWs despite treatment with low-dose aspirin (LDA) and unfractionated heparin (UFH) during her second pregnancy. Laboratory tests at her admission to our hospital (29 + 5/7 GWs) revealed hypercalcemia (serum calcium [Ca] of 10.7 mg/dL [normal: 8.8–10.1 mg/dL]) along with high serum levels of intact parathyroid hormone (iPTH) (81 pg/mL [normal: 15–65 pg/mL]). Additionally, urine tests revealed that the excretion of Ca was 453 mg/day and the Ca clearance to creatinine clearance ratio was 0.023. Therefore, she was finally diagnosed with PHPT at 29 + 6/7 GWs. She received hydration with 1,000 mL/day of maltose solution after PHPT diagnosis, and the Ca levels remained stable. However, she delivered a 1,056 g (−1.99 SD) female infant by cesarean section (CS) at 30 + 5/7 GWs due to non-reassuring fetal status (NRFS). Her baby experienced hypocalcemia and required temporary intravenous Ca supplementation. The endocrinologists and obstetricians recommended further workup and PHPT treatment, including parathyroidectomy, but the patient declined them and never visited our hospital.

**Figure 1:**
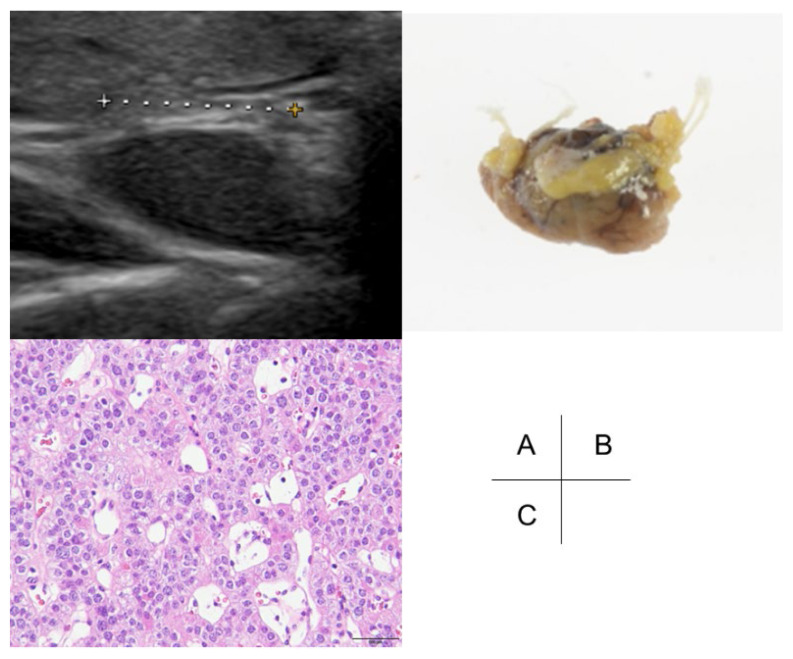
Ultrasound and gross and pathological results of the right superior parathyroid gland adenoma (A) The neck ultrasound assessment revealed an enlarged parathyroid gland (6 mm × 5 mm × 10 mm) posterior to the right lobe of the thyroid. (B) Gross findings of the resected right superior parathyroid gland adenoma. (C) Pathological results of the right superior parathyroid gland adenoma. Hematoxylin and eosin staining, ×100 objective. Cells with uniformly enlarged, round nuclei, densely proliferate in a pseudopapillary and trabecular pattern around a vascular fibrous stroma.

**Figure 2:**
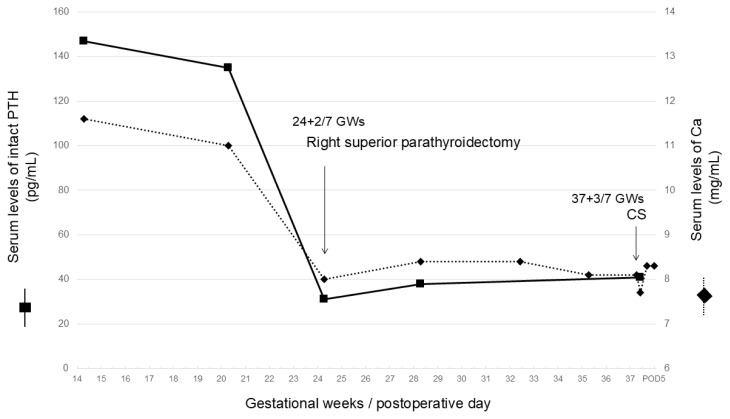
Clinical course of the patient (■) and (◆) indicate the serum parathyroid hormone (iPTH, pg/mL) and Ca (mg/mL) levels, respectively. iPTH: intact parathyroid hormone; Ca: calcium; CS: cesarean section; GW: gestational weeks; POD: postoperative day.

She was spontaneously pregnant and visited our hospital at 9 + 2/7 GWs. Combination therapy of LDA + UFH was commenced immediately due to low PS activity (31.0% at 9 + 2/7 GWs) and her FGR and IUFD history. Laboratory tests at 14 + 2/7 GWs revealed increased serum Ca (11.6 mg/dL) and iPTH (147 pg/mL) levels. Additionally, a neck ultrasound revealed an enlarged parathyroid gland (6 mm × 5 mm × 10 mm) posterior to the right superior lobe of the thyroid (Figure 1A). She reported no family history of endocrine tumors of the parathyroid, pancreas, or pituitary, indicating multiple endocrine neoplasia type 1 (MEN1). Our team, including obstetricians, endocrinologists, and otolaryngologists, recommended that the patient and her spouse reconsider parathyroidectomy. The following were the results of blood laboratory tests, except for serum Ca, before the operation: white blood cell counts: 7.7 × 10^3^/μL; red blood cell counts: 428 × 10^4^/μL; hemoglobin concentration: 10.4 g/dL; platelet counts: 28.2 × 10^4^/μL; serum levels of alanine aminotransferase: 7 U/L; aspartate aminotransferase: 11 U/L; lactate dehydrogenase: 135 U/L; total bilirubin: 0.4 mg/dL; direct bilirubin: <0.1 mg/dL; creatinine: 0.61 mg/dL; and blood urea nitrogen: 6.7 mg/dL. She underwent a single-gland (right superior parathyroid gland) parathyroidectomy at 24 + 2/7 GWs with informed consent (Figure 1B). The operation was completed by confirming significantly decreased serum iPTH (135 pg/mL at 20 + 2/7 GWs) levels during the procedure (31 pg/mL) (Figure 2). Finally, she was pathologically diagnosed with a right superior gland parathyroid adenoma (Figure 1C). Serum Ca and iPTH levels remained within the normal range postoperatively (Figure 2). She experienced no adverse events throughout the remainder of her pregnancy. She delivered a 2,136-g (−1.74 SD) female infant with Apgar scores of 8 (1 min) and 9 (5 min), and a pH of 7.367 in the umbilical cord blood by CS at 37 + 3/7 GWs due to previous CS. She and her baby were discharged 6 and 20 days after delivery without any clinical complications, respectively.

## DISCUSSION

PHPT is a relatively rare endocrine condition and the leading cause of hypercalcemia. Mild hypercalcemia is asymptomatic, but severe hypercalcemia causes severe symptoms, including impaired consciousness and coma (5).

The general population and women of reproductive age demonstrated PHPT prevalence rates of 0.15% and 0.008–0.05%, respectively (1, 2). A retrospective study revealed that 0.8% of patients with PHPT who required treatments were observed during pregnancy (6). However, the exact PHPT incidence during pregnancy is unknown, as many cases remain asymptomatic and untreated. Therefore, PHPT during pregnancy is very rare.

The most prevalent cause of PHPT is parathyroid adenoma (90%), followed by parathyroid hyperplasia (6%–9%) and parathyroid carcinoma (1%–2%) (1). MEN1 must be excluded when we encounter young patients with PHPT. In our case, a single-gland parathyroid adenoma caused PHPT. She exhibited no tumors other than in the parathyroid gland and reported no family history of endocrine tumors in the parathyroid gland, pancreas, or pituitary gland, indicating MEN1. Therefore, we ruled out MEN1.

PHPT during pregnancy causes pregnancy complications, such as preterm delivery, FGR, and IUFD, as well as neonatal complications, including hypocalcemia, tetany, and permanent hypoparathyroidism (7). PTH-related peptide (PTHrP) is released from the placenta and may act as a transporter of Ca and a modulator of fetal growth and development during pregnancy (8). Animal experiments reported that PTHrP concentrations in the placenta and amniotic fluid were decreased in growth-restricted and hypertensive rats (9). Maternal hypercalcemia may suppress PTHrP secretion from the placenta in pregnant women with PHPT, causing FGR. However, in this case, PTHrP levels were not measured in the placenta, amniotic fluid, fetus, or neonatal blood, so it is unclear whether the decrease in PTHrP levels was related to the occurrence of FGR or IUFD. Conversely, increased maternal serum Ca levels cause hypercalcemia in the fetuses, which induces fetal parathyroid gland suppression. The parathyroid glands of the fetuses remain suppressed after birth, inducing neonatal hypocalcemia either temporarily or permanently (8). Additionally, several case reports presented preeclampsia (PE) or HELLP (hemolysis, elevated liver enzymes, and low platelets) syndrome in pregnant women with PHPT (10, 11). Excessive PTH levels stimulate the renin-angiotensin-aldosterone system, inducing PE in pregnant women with PHPT (12). Indeed, our case of untreated PHPT during pregnancy caused FGR and IUFD in her first pregnancy, and preterm birth due to NRFS accompanied by FGR and neonatal hypocalcemia in her second pregnancy.

Moreover, a retrospective study revealed that a serum Ca concentration of ≥11.4 mg/dL increases the risk of pregnancy loss and IUFD (7). PHPT treatment has two ways: conservative and surgical. Conservative treatment is recommended in mild PHPT (serum Ca ≤ 11.4 mg/dL).. The conservative treatments include hydration and calcitonin, cinacalcet hydrochloride, and bisphosphonates administration. However, bisphosphonates are not recommended during pregnancy because they are harmful to fetal bone formation. Conversely, the curative treatment for PHPT involved surgical treatment, specifically parathyroidectomy. A previous systematic review that compared conservative and surgical management revealed that 47% of pregnant women with PHPT underwent parathyroidectomy during pregnancy, with 61% of those surgeries occurring in the second trimester. The review indicated that parathyroidectomy is preferable to conservative treatment, even for pregnant women with mild PHPT, as fetal and neonatal complications were less predominant in the surgical treatment group compared with the conservative treatment group (4). Generally, parathyroidectomy for PHPT during pregnancy should be performed in the second trimester because fetal organogenesis is incomplete in the first trimester, and prolonged hypercalcemia exposure worsens the prognosis for offspring (2, 13).

PHPT was diagnosed during the second pregnancy in our case, but she declined further PHPT follow-up and treatment after birth. In previous case reports, it has been suggested that parathyroidectomy yields favorable outcomes in pregnant women with PHPT, even when detected during pregnancy or in women with recurrent pregnancy loss (14, 15). Moreover, guidelines recommend that if parathyroidectomy is not performed during the ongoing pregnancy, it should be undertaken postpartum or prior to the next pregnancy (16). She should receive a parathyroidectomy before any subsequent pregnancies to avoid pregnancy and neonatal complications. However, she underwent a right superior parathyroidectomy in the second trimester of her third pregnancy. In our case, the patient underwent a single-gland parathyroidectomy in the second trimester and delivered a healthy live-born infant at term, although light-for-date (LFD). The pregnant woman did not smoke, and placental histology revealed no abnormal results related to LFD. Thus, the reasons why her newborn experienced LFD remain unclear, apart from PHPT and low PS activities.

Our case report indicated that pregnant women with PHPT, particularly those with a pregnancy complication history, should be considered for parathyroidectomy even during pregnancy. This case report provides useful information for clinical practitioners in perinatal medicine.
